# Data-Driven Network Analysis for Anomaly Traffic Detection

**DOI:** 10.3390/s23198174

**Published:** 2023-09-29

**Authors:** Shumon Alam, Yasin Alam, Suxia Cui, Cajetan Akujuobi

**Affiliations:** 1 Electrical and Computer Engineering Department, Prairie View A&M University, Prairie View, TX 77446, USA; sucui@pvamu.edu (S.C.); cmakujuobi@pvamu.edu (C.A.); 2 Department of Physics, University of Texas, Austin, TX 78712, USA; yfralam@utexas.edu

**Keywords:** anomaly detection, intrusion detection, machine learning, datasets, attack model

## Abstract

Cybersecurity is a critical issue in today’s internet world. Classical security systems, such as firewalls based on signature detection, cannot detect today’s sophisticated zero-day attacks. Machine learning (ML) based solutions are more attractive for their capabilities of detecting anomaly traffic from benign traffic, but to develop an ML-based anomaly detection system, we need meaningful or realistic network datasets to train the detection engine. There are many public network datasets for ML applications. Still, they have limitations, such as the data creation process and the lack of diverse attack scenarios or background traffic. To create a good detection engine, we need a realistic dataset with various attack scenarios and various types of background traffic, such as HTTPs, streaming, and SMTP traffic. In this work, we have developed realistic network data or datasets considering various attack scenarios and diverse background/benign traffic. Furthermore, considering the importance of distributed denial of service (DDoS) attacks, we have compared the performance of detecting anomaly traffic of some classical supervised and our prior developed unsupervised ML algorithms based on the convolutional neural network (CNN) and pseudo auto-encoder (AE) architecture based on the created datasets. The results show that the performance of the CNN-Pseudo-AE is comparable to that of many classical supervised algorithms. Hence, the CNN-Pseudo-AE algorithm is promising in actual implementation.

## 1. Introduction

Cyber-attacks are critical problems in today’s internet-based interconnected systems. Most connected systems, such as financial institutions, transportation systems, smart grids, and manufacturing processes, are vulnerable to cyberterrorism. The attacks could jeopardize an organization’s operations, safety, privacy, and stability. Online fraud has many forms, such as computer intrusions (hacking), economic espionage (theft of trade secrets), online extortion, identity theft, and internet-facilitated crimes. Recently, cyber-attackers hacked numerous interconnected systems, stole information and money, and perpetrated crimes. According to the US Federal Bureau of Investigation (FBI)’s *Internet Crime Report 2022* [1], the loss due to cybercrimes from 2018 to 2022 was USD 27.6 billion, whereas the loss in 2022 alone was USD 10.3 Billion. During these five years, the FBI’s Internet Crime Complaint Center (IC3) received over 3.26 million complaints. Apart from the report, there might be many other attacks not reported. Every year, the attack number grew many folds, affecting our personal lives and national security. With time, the attacks are becoming more and more sophisticated. According to the report [1], the top-five reported crimes were for tech support, extortion, non-payment/non-delivery, personal data breach, and phishing. The growing number of ransomware attacks crippled individuals as well as critical infrastructure sectors in 2022. This ransomware attack targeted the public health sector the most in 2022. According to the tech company Astra’s prediction, the global cost of cybercrime will reach USD 8 trillion in 2023 [2]. It is necessary to identify system vulnerabilities to minimize loss from these cyber-attacks. Many vulnerabilities can be found due to the software’s weakness, IT policies, human errors, and network architecture. 

An intrusion detection and protection system (IDPS) is critical to any network infrastructure. There are two types of IDPS. One is host-based, and the other is a network-based system. A network-based IDPS is a system that usually sits at the boundary of the internal and external networks (there are various ways to implement it), and it is the primary system to detect and block cyber-attacks. A classical network-based IDPS works based on the attack signatures but is useless for zero-day and sophisticated attacks. These IDPSs are network vendors’ firewalls (software or independent system). Due to the inability to detect sophisticated cyber-attacks, vendors like Palo Alto developed the first-ever smart firewall based on machine learning (ML) algorithms in 2020 [3]. ML comes in various flavors: supervised learning (SL), unsupervised learning (UL), and semi-supervised learning (S-SL). ML algorithms require training on network traffic for intrusion detection. SL requires “labels” for training comparable to the classical IDS system based on signature detection. S-SL algorithms also need a small portion of labeled data with lots of unlabeled data to train a predictive model. UL methods do not require labeled data but a good dataset for developing predictive models. Because UL does not need labeled data, creating IDS using UL methods is more attractive than other learning methods. Researchers used various UL algorithms for IDS development and used NSL-KDD datasets predominately to measure the performances. In developing IDS using ML, a useful, realistic network dataset is required to train the ML-based detection engine. Our research community uses public datasets with various limitations, including limited attack scenarios, unrealistic traffic, etc. We need a dataset created from realistic network traffic considering various cyber-attack strategies with diverse background traffic in a full network topology. The dataset also has to be large enough for training and testing.

To overcome various limitations of the public network datasets, we have developed various network raw data or datasets in this work. We have extended our work [4], considering the various realistic cyber-attacks, and developed testbeds to produce those cyber-attacks in a complete network topology using Spirent’s CyberFlood CF20 [5]. We have created a testbed with an attack network and a target network with a DMZ, which included a web server, a DNS server, and a mail server. A firewall has been added between the attack and the target network. The attack network includes tools based on the Linux and Windows OS and the CyberFlood CF20, an industry-standard traffic emulator. CF20 can create hundreds of zombie attackers with various background traffic. The emulator follows the full protocol stacks to emulate the traffic realistically. It also has a library with cyber-attack scenarios updated regularly by Spirent Communications. With the library’s help, users can recreate the most current cyber-attacks. In this work, we follow the unified attack classes: communication passive, communication active, host class, and application class. We create most attacks using the CyberFlood emulator. The work has produced raw data or datasets for all the attack classes. The raw data development includes generating attack and benign traffic from the attack network to the victim/target network and capturing the traffic flows at the target using TCP-dump commands or the Wireshark tool. The captured raw data (attack traffic, benign traffic) are then converted to datasets, e.g., CSV files. 

In [4], we measured the performance of some standard supervised ML algorithms using the PVAMU-DDoS2020 dataset. The algorithms are Gaussian naïve Bayes (GNB), quadratic discriminant analysis (QDA), random forest (RF), iterative dichotomizer 3 (ID3), adaptive boosting (AdaBoost), multilayer perceptron (MLP), and K-nearest neighbor (KNN). In [6], we developed an unsupervised ML algorithm, the CNN-Pseudo-AE model, and measured the performance of anomaly traffic detection using the same dataset. In addition to the generation of new raw data/datasets development in a realistic approach, in this paper, we also compared the performances of the classical supervised ML [4] and our developed UL algorithm, CNN-Pseudo-AE, to justify that the proposed CNN-Pseudo-AE is a good candidate for actual realization since UL based solution is more attractive in the actual implementation. 

The current work has produced the following contributions:

1. Development of raw data or datasets considering realistic cyber-attacks for network intrusion detection applications.
2. Publication of data/datasets online for the research community [7].
3. Identification of the CNN-Pseudo-AE unsupervised algorithm as a good candidate for actual implementation for anomaly traffic detection.

The remainder of the article is organized as follows. Section 2 includes the material and methods, including the literature review of the public datasets, a description of network vulnerability, the process of dataset development, and the testbed description. Section 3 includes the result and comparisons between the supervised and unsupervised ML algorithms. Finally, Section 4 concludes the article.

## 2. Materials and Methods

### 2.1. Literature Review

The cyber-attack patterns change often, and detecting a zero-day attack using firewalls based on the signatures is impossible. Because of the inability to detect sophisticated attacks, network vendors are working to develop firewalls with machine learning (ML) techniques. For attack or anomaly traffic detection, ML requires training and testing of a system based on network traffic. Most vendors use proprietary datasets for their development, but the research community uses open/public datasets for their research activities. Various public datasets are available for developing network intrusion detection systems (IDS), and many datasets are unsuitable due to proprietary issues. Many datasets are generated by removing various network traffic features (anonymized dataset, e.g., packet inter-arrival time, flags, etc.). Other datasets are created using synthetic methods that do not include realistic attack scenarios, which are not generated via complete protocol stacks. We identify that the Canadian Institute of Cybersecurity’s CICIDS2017/19 dataset [8] is the most comprehensive dataset developed so far. The institute used a complete network topology for generating various attack traffic with benign background traffic for several days and captured the traffic (inbound–outbound) for analysis using CICFlowMeter [9]. Still, CICIDS 2017/19 is not free of limitations. It has limitations in user profiles, traffic diversity, and simulation methods. The background traffic was simulated for 25 users only for five different protocols (HTTP, HTTPS, FTP, SSH, and email protocol). We do not know the network load and other parameters from their work. Table 1 includes many existing public datasets and their limitations based on literature reviews [10,11,12,13,14,15,16,17,18,19,20,21,22,23,24]. The key issues are listed below:

1. Size of the dataset;
2. Lack of diverse traffic;
3. Lack of diverse attacks;
4. Unrealistic scenarios;
5. Synthetic development process;
6. Produce traffic by simulation;
7. Network topology.

**Table 1:** Common public network dataset.

Name of Dataset	Description	Limitations
DARPA (Lincoln Laboratory 1998-1999) [10]	The first available dataset for network intrusion detection applications.	The dataset is very old and is considered outdated. This dataset does not include realistic network traffic and contains irregularities. The developer used synthetic and simulated background traffic. Attacks were implemented via scripts and programs from different sources [11]. It does not have any validation of synthesized traffic to real traffic. It is too clean and predictable [12].
KDD99/KDDCup99 (University of California, Irvine 1998-1999) [13]	The dataset was extracted from the DARPA’99 dataset. It is widely used to evaluate detection methods [14]. The dataset includes the DoS, user-to-remote (U2R), remote-to-local (R2L), and probing attacks. It has 19% benign traffic, 74.41% DOS, 1.33% probe, 0.07% U2RL, and 4.68% R2L attack traffic [15].	The dataset has duplicated samples. It does not have diverse benign and modern attack traffic. It can also be considered outdated. It has unbalanced data samples in each class [15].
NSL-KDD (2009) [16]	It was developed from KDD99. Duplicates were removed from the KDD99 dataset [15].	This dataset still lacks newer attack types. It still contains imbalanced samples in each class [15].
Gure-KddCup [17]	The dataset was also developed from the DARPA 1998 dataset by adding additional payloads and other features related to the traffic flows, such as IP address and port number [18].	This dataset suffers the same issues as that of the DARPA original dataset. It is a lack of diverse attacks and benign traffic [18].
Cyber Defence Exercises (CDX) (United States Military Academy 2009) [19]	The dataset was developed from the system logs. It has buffer overflow attacks. Traffic was divided into intrusion traffic and normal traffic [20].	It is heavily anonymized (not all traffic attributes are available in the dataset) [20].
Kyoto (Kyoto University 2009) [21]	The dataset was developed using honeypots. The traffic was collected daily between 2006 and 2015 [20].	Since it was created from the traffic at the honeypots, they may not represent realistic attacks. The type of attack is not well defined [20].
Aegean WiFi Intrusion Dataset (AWID) [22,23]	It contains a rich blend of normal and attack traffic on 802.11 networks (WiFi). The dataset is developed for intrusion detection for wireless networks. The traces collected in AWID are real and extracted from the actual utilization of a dedicated WEP-protected 802.11 network [22].	The dataset contains 156 features. The data is noisy, and the features are misleading. It also includes uncategorized samples [22].
CICIDS2017 [8]	This dataset was developed by the Canadian Institute for Cybersecurity using full network configurations. It has more current types of attacks. It includes SQL injections, brute force attacks, and flooding attacks. [20].	Attacks were made mainly using simulation tools, and it lacked of realistic scenarios. A highly imbalanced dataset creates low accuracy and a high false positive rate when applied to ML tools. It also includes duplicate samples [20].
UNSW-NB15 [24]	The dataset was developed by the Cyber Range Lab of the Australian Centre for Cybersecurity. It includes 49 features. The dataset consists of attacks such as fuzzers, backdoors, shellcode, DoS attacks, worms, generic attacks, reconnaissance attacks, exploits, and analysis attacks. The dataset is more comprehensive than KDDCUP99, and it is an improved version of NSL-KDD. It has limited modern attack scenarios [25].	The dataset was created synthetically [25].
ISCX IDS 2012 [8]	The dataset was developed at the University of New Brunswick (UNB). The dataset includes normal and attack traffic such as DoS, DDoS, and brute force SSH attacks. The dataset was created using a dynamic approach which allowed the creation of new datasets [25].	The dataset was prepared from unrealistic attack scenarios. Actual background traffic for the recipient device was not included [26].
ASNM-CDX (2009) [27]	This dataset was created from the CDX network traffic data. The dataset includes 5772 samples, each with 875 + 1 + 1 features. It consists of buffer overflow attacks only [27].	The dataset lacks traffic diversity since it consists of buffer-overflow attacks only [28].
Lawrence Berkeley National Laboratory (LBNL, 2004–2005) [29]	This dataset was created at the Lawrence Berkeley National Laboratory (LBNL) between 2004 and 2005. The payloads are anonymized due to privacy issues.	Due to the privacy issue, the dataset is heavily anonymized.
Czech Technical University-13 (CTU-13, 2011) [30]	The dataset was developed at Czech Technical University in Prague (CTU). It considered botnet scenarios in addition to normal and background traffic [30].	The dataset is designed only for botnet detection [30].
University of Twente (Twente, 2009) [31]	The dataset includes 14 million flows and more than seven million alerts. The critical data from the packet headers and payloads are anonymized [31].	The traffic was captured from the honeypots. The traffic was unrealistic, and more advanced attacks were missing due to honeypot use [31].
CAIDA (Center of Applied Internet Data Analysis 2000–2016) [32]	The dataset is very specific to a particular attack, e.g., DDoS or internet activity [20]	The dataset does not accurately represent the different possible types of attacks. Datasets are specific to particular events or attacks and are anonymized with their payload, protocol information, and destination [20].
DEFCON (2000–2002) [26]	The datasets were developed for intrusion modeling competitions. It focused only on intrusions and attacks and lacked normal background traffic) [26].	The dataset does not have normal background traffic. It is not usually used in the intrusion detection research fields [26].
UMASS (University of Massachusetts 2004–2018) [33]	It contains traffic data such as Tor traffic data, Gateway Link 3 Trace data, web requests, and response data [34].	It was generated using a single TCP-based download request attack scenario. The dataset does not help test IDS and IPS techniques due to the lack of variety of traffic and attacks [34].
DDoS2019 (CIC) [34]	The dataset is more comprehensive than any other public dataset [34].	Simulation tools are used for DDoS attacks [4].

### 2.2. Network Vulnerability

Network vulnerability comes in two ways concerning private and public networks. In a classical network architecture, a network behind security, such as firewalls, is considered a private or internal network, and anything outside the firewalls is a public network. An enterprise network is vulnerable to both internal and external attacks. Internal threats are linked to employees who carry out attacks by exploiting their inherent privileges. An enterprise network is more prone to external attacks due to human factors such as misconfigurations, users in the phishing trap, weak IT management, etc. In this article, we focus on external attacks since most cyber-attacks originate from remote sources. A persistent attack is more critical compared to a non-persistent attack. Persistent attackers obtain network access, remain undetected, and even remove their tracks and presence. At the same time, the non-persistence attack is a one-time attack and tries to steal information. Cyber-attackers use various techniques to attack. Some attacks are nothing but gathering information in the beginning. Some common techniques for gathering information regarding the hardware, network configurations, and user information are reconnaissance, port scanning, and ping sweeping. Attackers exploit the network vulnerability to steal information by creating backdoors and obtaining escalate privileges, deny services and crash services, and even modify or alter information via scripting or privileged access. Zippy Falco et al. [35] defined cyber-attacks in four classes: Communication Active, Communication Passive, Host, and Application. These four classes can be divided into sub-types based on the DARPA data classifications. Based on the four classes and DARPA classification [10], a unified attack model is given in Table 2. It also includes the class description.

The communication-passive-class attack model is about gathering system information/reconnaissance process for plotting cyber-attacks by discovering network vulnerability. The process does not harm the targeted network, but the discoveries of the vulnerability inspire the attackers to perform communication-active/host/application-class-based attacks. Communication-active-class attacks damage the network services. Such attacks are denial of service (DOS) or distributed denial of service (DDoS). These attacks can crash any service by over-utilizing the service process and exhausting the bandwidth usage. Host and application-class-based attacks are more sophisticated. These attacks are basically malware-driven attacks. Attackers exploit the system or application loopholes to create backdoors and, in the process, obtain privileged access to the systems. These attacks are more dangerous since through this process, attackers can steal information and can engage in various criminal activities, such as asking for ransom. This is why an enterprise must audit its network vulnerabilities often, such as by penetration testing.

### 2.3. Dataset Development

ML-based solutions need a dataset for training and prediction. A good dataset is a primary need for ML-based solutions. A good dataset could be defined for network intrusion or anomaly traffic detection by considering large data size; diverse benign and attack traffic based on open systems interconnection (OSI) layers 3, 4, and 7; and diverse traffic protocols. The dataset must be developed based on realistic network scenarios where traffic follows the standard protocol stacks. The primary challenge is the unavailability of labeled traffic datasets for various network activities and attack cases. Existing datasets have various limitations, and some are outdated, as mentioned in Table 1; hence, in this work, we propose developing realistic datasets for network intrusion detection considering various network attack classes given in Table 2 and diverse benign traffic considering various network protocols in a full network topology. It is challenging to consider and create realistic scenarios in a lab setting. Thus, to overcome the issues of generating real network traffic for various attack scenarios, we have used Spirent’s CyberFlood CF20, an industry recognized traffic emulator/tester for testing network vulnerability.

### 2.4. Testbed Description

**Figure 1:**
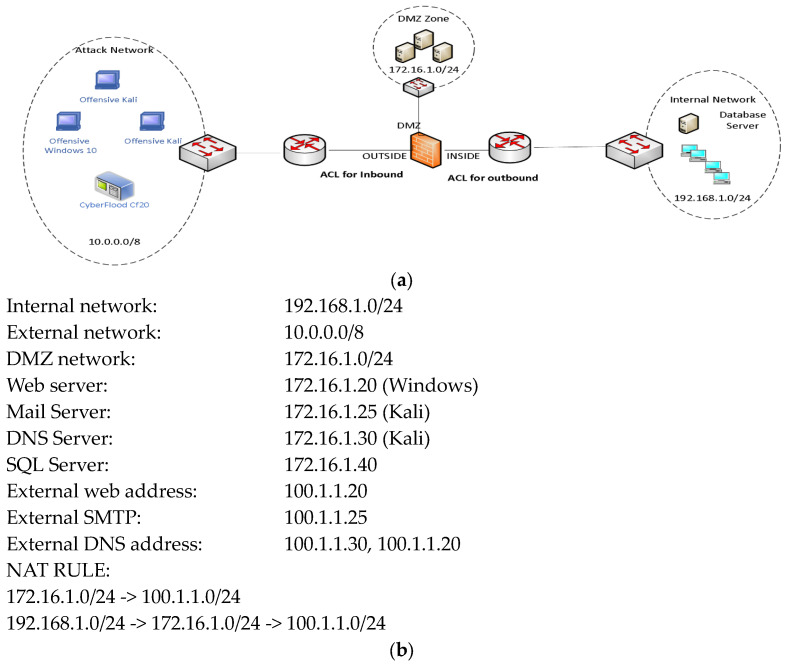
(**a**) Testbed for network traffic (benign and attack) generation. (**b**) Network Details for (**a**).

Figure 1a shows our baseline network architecture. It includes attack and victim networks (internal and DMZ). The network is implemented considering classical network architecture in which a DMZ includes a web server, mail server, SQL server, and DNS server for providing services to external and internal requests. The attack network includes CF20, Kali, and Windows clients. The DMZ includes servers (Kali, Ubuntu, and Windows OS). The Cisco ASA 5505 firewall is configured to translate any legitimate requests for web, DNS, mail, and database services from external and internal sources. For both cases, the firewall uses the NAT protocol. We have used one Catalyst 9300 for implementing three switches via VLAN methods. VLAN 10 is for the internal network, VLAN 20 is for the external network, and VLAN 30 is for the DMZ network. Each port’s throughput is 1 Gbps. Port 48 is a mirror port for the DMZ network. The network details are given in Figure 1b.

**Figure 2:**
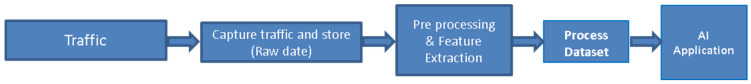
Work Process.

Figure 2 shows the basic methodology of the network-traffic dataset development process. The process includes the following steps:

- Emulate benign and attack scenarios and generate traffic.
- Capture raw traffic.
- Study raw traffic and feature extraction.
- Develop/Process dataset.
- Analyze datasets with ML/DL

Following Table 2, we used CF20 and Linux tools to develop network datasets or raw data for four attack classes. We emulated various network attack classes, generated traffic, and captured the raw data. Various subclasses are described below.

A. Probing Dataset (Communication Passive Class):

Probing is a communication-passive-class attack. Assuming we have pre-knowledge of the public IP addresses of the victim networks, we used NMAP commands to probe the system/server information along with HTTP background traffic from CF20. Probing was performed to external IP addresses 100.1.1.20-30. Table 3 includes all the probing types:

B. DDoS Dataset (Communication Active Class)

DDoS is a communication-active-class attack. There are two main types of DDoS attacks: volumetric DDoS (VDDoS) and protocol DDoS (PDDos) attacks, based on OSI layers 3 and 4, respectively. Volumetric DDoS attacks are based on ICMP attacks, whereas PDDoS attacks are based on the UDP and TCP protocols. We have completed both types of attacks, using CF20 from the attack network to the DMZ network, bypassing the firewall to create up to 1 Gbps attacks and capturing the bi-directional traffic from the DMZ switch using TCPdump or the Wireshark tool. The captured file size was from 10 to 17 GB in pcap format.

We generate VDDoS attacks based on ICMP flooding. This attack is based on open systems interconnection (OSI) layer 3. ICMP is generally used for messaging between two devices. When the number of ICMP-based requests is in the millions, the process saturates the network bandwidth and wastes computing power. This process is simple but can quickly bring down perimeter devices or saturate bandwidth. ICMP has various types. In the dataset, we included ICMP Type 0 (echo reply), 3 (destination unreachable), 8 (source host isolated), and 11 (destination network unreachable for type of service) floods.

We generate PDDoS attacks using the OSI layer 4 protocols, UDP and TCP. UDP-based attacks or UDP packet floods saturate network bandwidth and bring down network services. The attacks include a rapid succession of UDP datagrams (garbage payload) with spoofed IPs to a server using different ports (53, 123). They force the server to respond to ICMP traffic. Both ingress and egress bandwidth are saturated with this attack. We have considered a UDP flood to port 53 (domain name service (DNS)) to bring down the DNS service. The TCP-based attacks are SYN Flood, ACK Flood, and RST Flood. These attacks exhaust the states and cripple a server’s ability to respond to legitimate requests. The SYN flood saturates and prevents the completion of the TCP three-way handshake between the client and the server. The target server’s communications port becomes a half-open state. In this work, we have used ports 25, 80, and 443 for the SYN flood. The TCP ACK flood occupies the server with the flooded junk data and blocks the server from providing service to other users by slowing down or crashing the target using junk data. The targeted server uses so much computing power to respond to each ACK packet received that it cannot serve legitimate users. We have used ports 25, 80, and 443 for the ACK flood. The TCP-RST flood saturates the network bandwidth and resources on stateful devices. When millions of RST packets are sent continuously, the process can bring down the stateful defense. This can create unexpected issues and behavior. We have used ports 25, 80, and 443 for the RST flood. In this work, we also used XMAS Tree Flood, a PDDoS type of attack, using the CF20 emulator. A Christmas tree attack sends a large number of packets with all the options turned on so that any protocol can be used. This kind of packet requires more processing by end devices and routers. If a large number of these packets are sent, they keeps the receivers busy in processing, and it is nearly impossible for it to perform other tasks. Thus, the receivers deny the legitimate traffic.

We also used benign traffic to develop the DDoS dataset. Benign traffic included HTTP1.0, HTTP1.1, HTTPs, FTP, SSH-Login, Telnet, SMTP, POP3, RTSP, SIP, BitTorrent, Exchange, CIFS/SMB, IMAP4, SSH, and Facebook traffic. Table 4 and Table 5 include the attack configurations and various outcomes for VDDoS and PDDoS attack scenarios.

C. Host & Application Class

**Figure 3:**
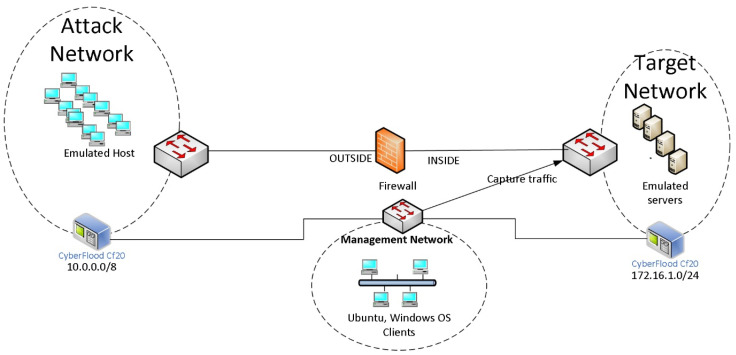
Testbed for cyberattack and malware attacks from attack network to target network.

The host class includes the unauthorized access from remote to local (R2L) machine attacks and unauthorized access to root (U2R), whereas the application class includes the R2L attacks only. Cyberattacks have now become very sophisticated, and it is challenging to distinguish the R2L from the U2R and vice versa. These attacks look like regular/benign traffic, but they take control of the host systems/applications by gaining access. It is challenging to produce actual traffic characteristics of these attacks. Considering this fact, we investigated the Host and Application attack classes using the real cyberattacks that occurred from 2019 to 2022. To emulate these attacks, we modified our testbed, given in Figure 3. We took advantage of CF20′s capability of emulating actual network/cyberattacks. Its library has up-to-date attack models which are used to create the actual host and application attacks.

The following attacks were emulated using CF20 in our sandbox environment.

- Major cybersecurity attacks that occurred during January–June 2022
- Major cybersecurity attacks that occurred during January–December 2021
- Major cybersecurity attacks that occurred during January–December 2020
- Major cybersecurity attacks that occurred during January–December 2019
- Major malware attacks that occurred during January–June 2022
- Major malware attacks that occurred during January–December 2021

The attacks were performed in two different categories. They were (i) general cyberattacks and (ii) malware attacks. Attack profiles were built to send realistic, stateful attack and malware traffic using Cyberflood’s TestCloud. The TestCloud includes an attack library that is automatically updated, providing thousands of the latest, most relevant attacks to choose from. Figure 3 shows a simple logical testbed for traffic generation and collection, while Table 6 includes the attack types and the collected traffic summary. The attack network (10.0.0.0) had thousands of emulated attack hosts in a flat network, and the target network (172.16.0.0) included various emulated servers such as DNS, POP3, Webserver, SQL Server, etc. The testbed included a firewall without any ACL rules so that communication could be established between the attacking hosts and the emulated servers, and bidirectional traffic could be captured by sniffing traffic from a switch port.

Some of the attack techniques used for attack scenarios are given below:

- Remote code execution
- Unauthenticated attack
- Cross-site scripting
- Arbitrary code execution in the context of the application
- Exploitation by remote code execution in the security context of the system
- Buffer overflow attack
- Authentication bypass attack
- Unrestricted file upload and remote code execution
- WordPress contact form entries plugin stored cross-site scripting
- Buffer overflow attack in Oracle MySQL NDB cluster component
- SQL injection
- Arbitrary code execution on the target system
- Execution of arbitrary script code in the target user’s browser
- Crafted request to the target server
- Stack-based buffer overflow
- Heap buffer overflow
- Out-of-bounds read and write
- Denial of service by terminating the server due to an unhandled exception
- Arbitrary command execution attack

Some of the malware types used for attack scenarios are given below:

- Backdoor that targets the Windows platform
- Ransomware that targets the Windows platform
- Worm that targets Linux operating systems
- Trojan malware
- Trojan—password stealer
- Backdoor.MSIL.Crysan Trojans
- Data exfiltration
- Trojan—downloader
- Malicious_confidence_90%
- Backdoor.DCRat

Table 7 includes all the raw data captured for various scenarios created in this work. Since R2L and U2R traffic are difficult to distinguish, we have labeled the cyberattacks as R2L/U2R and the malware attacks as Application attacks.

D. Benign Mixed Traffic

To develop a dataset for benign traffic, we have emulated network traffic using DNS, HTTP, IMAP4, POP3, SMTP protocols, and RDP, SNMP, SSH, Syslog, uTorrent, and Exchange applications to create benign mixed traffic. We emulated Netflix and Facebook traffic using CF20 for streaming and social networking traffic. The traffic is captured in pcap format. A traffic request is sent from 10.0.0.0 to 172.16.0.0 on the server network. Table 8 includes the various traffic types for developing datasets with benign traffic. The raw data and the datasets are available at the SECURE Cybersecurity portal [7]. They are freely available to the research community.

## 3. Results: Performance Comparisons of SL and UL-Based Algorithms’ Anomaly Traffic Detection

Our work has produced various data/datasets considering realistic traffic and attack scenarios. In this sub-section, we have focused on DDoS attacks to compare the performance of some standard SL models and our developed UL model. Zombie attackers can create millions of requests (DDoS attacks) to any service, exhaust the bandwidth, and increase response time for legitimate requests. The attack can crash any network service. Often, it is complicated to separate attack traffic and legitimate traffic since the attack requests also follow the TCP/IP protocol stack. A conventional firewall is not capable of identifying this kind of attack. ML can be used to identify DDoS attacks based on traffic attributes. ML-based systems can be trained on the DDoS traffic and used to identify anomaly traffic. In this work, we compare the performance of detecting DDoS traffic between the classical supervised ML [4] and our developed unsupervised ML algorithms (CNN-Pseudo-AE model [6]) using the PVAMU-DDoS-2020 dataset. The SL model produces the best outcomes since the models use the labeled datasets, whereas the UL models do not use labels. Our goal is to compare the performance of our model to that of the SL models to show how good our model is. Below, we include a short description of the PVAMU DDoS2020 dataset.

### Description of PVAMU DDoS2020 Dataset

The PVAMU DDoS2020 dataset has over 4.5 million traffic flows [4]. The captured raw data were processed into a labeled dataset using CICFlowMeter [9]. This tool can convert the PCAP file to a CSV format with 83 attributes/features for each traffic flow. Some attributes, for example, are flow duration, total number of packets in the forward direction, flow bytes/s, mean time between two packets sent in the flow, etc. For a detailed description of the attributes, readers are suggested to refer to [34]. The PVAMU-DDoS2020 dataset includes the ICMP-based OSI-layer 3 attacks that have ICMP Types 3, 8, and 11; UDP-based OSI-layer 4 attacks at port 53 (DNS); TCP-based layer 4 attacks that include SYN attacks at ports 25 (SMTP), 80 (HTTP), and 443 (HTTPS service); Xmas tree attacks; and benign traffic from 100 users’ profiles with mixed traffic that includes HTTP1.0, HTTP1.1, HTTPS, FTP, SSH-Login, Telnet, SMTP, POP3, RTSP, SIP, BitTorrent, Exchange, CIFS/SMB, IMAP4, SSH, and Facebook traffic. Overall, the dataset has 67.33% DDoS attack traffic and 32.67% benign traffic. Each traffic flow in the dataset has 83 features/attributes. The first seven features—low ID, source IP, source port, dest-port, protocol, and timestamp, are not needed for the training and testing. The rest of the 76 attributes are useful for ML applications.

In our previous work [4], we used the PVAMU-DDoS-2020 dataset, reduced the attributes to 14 features using PCA analysis, and utilized the new dataset for the performance measurement of some classical supervised ML algorithms. Table 9 includes the measurement outcomes based on the standard evaluation metrics: precision (Pr), rcall (Rc), and F-measure (F1 score) [4].

Since the attacking nature changes with time, it is challenging to create an up-to-date labeled dataset to be used with supervised ML algorithms, and thus it is more desirable to use unsupervised ML algorithms to identify anomalous traffic. In our previous work [6], we developed a CNN-based pseudo auto encoder (CNN-Pseudo-AE) unsupervised algorithm and measured the performance using the PVAMU-DDoS-2020 dataset. Table 10 includes the performance of detecting anomaly traffic for the CNN-Pseudo-AE algorithm [6].

In Table 9, the best outcomes with respect to the evaluation metrics, precision, recall, and F1 score were for Gaussian naïve Bayes (GNB), iterative dichotomizer 3 (ID3), adaptive boosting (AdaBoost), and K-nearest neighbor (KNN), but the AdaBoost and KNN algorithms took a long time to train and test the predictive models. GNB and ID3 algorithms’ performances were comparable even for the execution time. The ID3 and the AdaBoost models achieved 100% detection accuracy, which raises a red flag. The outcomes could be issues of the complex and overfitted models and even data leakage due to the extensive training and testing data sizes. If we compare Table 9 and Table 10, we can see that the performance of the CNN-Pseudo-AE algorithm (trial model #4) is very close to the performance of the GNB and KNN. The precision, recall, and F1 scores are 0.998, 0.998, and 0.998, respectively, for the GNB and KNN models. The execution times for the GNB and KNN models are 13.37 and 15,950.61 s, respectively. The KNN model took a long time to train and test due to the fact that KNN is a slow algorithm. The training time for the CNN-Pseudo-AE model on the same dataset is 5535.19 s, and the testing time is 744.58 s. The difference occurred because we utilized only the 14 features (reduced by PCA analysis) with the supervised algorithms [4], whereas, for our unsupervised learning, we did not reduce the features. We used all key 76 features plus 5 null features to create a 9 × 9 matrix to apply the CNN concept in a 2D structure. Overall, the performance of our unsupervised ML algorithm with respect to the Pr, Rc, and F1 metrics is outstanding. The accuracy is approximately 99.3%. This indicates that our CCN-based pseudo-AE algorithm is a good candidate for the actual implementation of anomaly traffic detection.

## 4. Conclusions

In this work, we developed raw data/datasets for various realistic cyber-attack scenarios that include probing attacks, DDoS attacks, remote to local (R2L, unauthorized access from a remote machine), and user to root (U2R, unauthorized access to local superuser (root) privileges) attacks. The attacks were created considering real traffic using Spirent’s CyberFlood CF20 traffic emulator to overcome the various limitations in public datasets. Our developed data contains diverse cyber-attacks and background traffic. Machine-learning-based solutions need suitable datasets to improve training and testing accuracy. The newly developed network data will benefit the further development of intrusion detection systems (IDS) using machine learning algorithms. Further, we compared the performance of anomaly traffic detection of some standard classical supervised ML and our unsupervised CNN-Pseudo-Autoencoder (AE) model. We showed that the performance of our developed CNN-Pseudo-AE model is very close to that of the supervised models. The accuracy of the CNN-Pseudo-AE model is about 99.3%. Because of its very high accuracy, with fine-tuning, this model can be considered for actual implementation.

## Figures and Tables

**Table 2:** Unified attack model.

Class	Description	Subtype (Per DARPA Dataset)
Communication Passive	Gathering information without active damage	Probe
Communication Active	Active attacks to damage the system	DOS, DDoS
Host	Attacks on a host by installing malicious code into it	Remote to local (R2L): unauthorized access from a remote machine
		User to root (U2R): unauthorized access to local super-user (root privileges)
Application	Attacks that target a given application aiming at executing malicious code by penetrating interfaces	R2L

**Table 3:** NMAP probing type.

NMAP PROBING TYPE
NMAP host scan
Nmap ping scan
TCP SYN scan
TCP connect scan
TCP ACK scan
TCP window scan
UDP scan
Null scan
FIN scan
XMAS scan
OS-fingerprinting

**Table 4:** VDDoS attack summary.

Attack Network IP Address	Victim Network	VDDoS Test Packet	Type	Duration (Min)	File Size, GB (Only Attack)	File Size, GB (Attack with Background Traffic)
10.0.0.101-200	172.16.1.0 Server Address: 172.16.1.100-15	Normal	ICMP Type 0, 3, 8, 11	3 to 6	11–16	10–12 GB
		Malformed	TCP Flood UDP Flood (port 53, 123)	3 to 6	16–17	10–12 GB
		Raw	IP 54 IP 58 IP 74		16–17	10–12 GB

**Definition of Packets:** Normal—normal packets are defined by the standard protocol; Malformed—a malformed packet means the protocol dissector (e.g., Wireshark) cannot further dissect the packet’s contents. Raw—raw IP protocol packets are defined as IP packets with the IP protocol field set.

**Table 5:** PDDoS attack summary.

Attack Network IP Address	Victim Network	PDDoS Test (Connection-Oriented) Load 500 Mbps	File Size, GB (Only Attack)	File Size, GB (Attack with Background Traffic)
10.0.0.101-200	172.16.1.101-115	SYN Flood–Port 25 SYN Flood–Port 80 SYN Flood–Port 443	7–10 GB.	4–6 GB
		ACK Flood–Port 25 ACK Flood–Port 80 ACK Flood–Port 443	7–10 GB.	4–6 GB
		RST Flood Type-1 Port 25 RST Flood Type-1 Port 80 RST Flood Type-1 Port 443	7–10 GB.	4–6 GB
		RST Flood Type-2 Port 25 RST Flood Type-2 Port 80 RST Flood Type-2 Port 443	7–10 GB.	4–6 GB
		XMAS Tree Flood-Port 25 XMAS Tree Flood-Port 80 XMAS Tree Flood-Port 443	7–10 GB.	4–6 GB.

**Table 6:** Attack and captured traffic summary.

Attack Profile	Captured File Size	Duration	Number of Attack Scenarios	MITRE ATT&CK Tactic/Techniques Summary
Cyberattack, January–June 2022	18.9 MB	11 min 23 s	129	9/35
Cyberattack, January–June 2021	236 MB	2 h 24 min	2714	11/125
Cyberattack, January–June 2020	80.1 MB	41 min 18 s	399	11/76
Cyberattack, January–June 2019	129 MB	1 h 1min	975	10/91
Adv. Malware attack, January–June 2022	1.95 GB	23 h 39 min	4732	10/21
Malware attack, January–December 2021	5.12 GB	70 h 1 min	9775	12/93

**Table 7:** Raw data description.

Attack Class Subtype	Raw Data File	Description
R2L	Cyberattack, January-June-2022	Emulated Attack traffic for January–June 2022
R2L	Cyberattack, 2021	Emulated Attack traffic for 2021
R2L	Cyberattack, 2020	Emulated Attack traffic for 2020
R2L	Cyberattack, 2019	Emulated Attack traffic for 2019
Application	Malware attack, 2021	Emulated Traffic includes malware attacks for 2021
Application	Adv_malware_attack_Jan-Jun_2022	Traffic includes adv malware attack scenarios for January–June 2022

**Table 8:** Benign Traffic Types.

Traffic Group	Traffic Type
Mixed Traffic	DNS, HTTP, IMAP4, POP3, SMTP, SSH, RDP, SNMP, Syslog, uTorrent, Exchange
Streaming	Netflix, YouTube, Skype
Social	Facebook

**Table 9:** Comparison of ML algorithms for DDoS detection [4].

Algorithm	Precision (Pr)	Recall (Rc)	F1 Score	Execution (second) (Training + Testing Time)
Gaussian Naïve Bayes (GNB)	0.998	0.998	0.998	13.37
Quadratic Discriminant Analysis (QDA)	0.811	0.554	0.534	13.72
Random Forest (RF)	0.893	0.873	0.863	14.98
Iterative Dichotomizer 3 (ID3)	1	1	1	18.25
Adaptive Boosting (AdaBoost)	1	1	1	3057.83
Multilayer Perceptron (MLP)	0.893	0,873	0.863	3967.68
K-Nearest Neighbor (KNN)	0.998	0.998	0.998	15,950.61

**Table 10:** Performance of CNN pseudo autoencoder algorithm [6].

Trial-Model No.	Precision	Recall	F1 Score	Accuracy	Train Time (second)	Test Time (second)
1	0.9811	0.9989	0.9899	0.9898	5147.85	725.67
2	0.9831	0.9989	0.9909	0.9869	3327.62	691.75
3	0.9775	0.9989	0.9881	0.9880	6382.35	871.37
4	0.9871	0.9989	0.9930	0.9929	5535.19	744.58
5	0.9844	0.9992	0.9918	0.9917	8109.15	934.18
6	0.9813	0.9989	0.9900	0.9899	4450.43	756.24

## Data Availability

Data is available in [7].
